# P-1286. In vitro Activity of Cefiderocol against Carbapenem-nonsusceptible Acinetobacter baumannii-calcoaceticus complex, Including Molecularly Characterized Clinical Isolates, Causing Infections in United States Hospitals (2020–2024)

**DOI:** 10.1093/ofid/ofaf695.1474

**Published:** 2026-01-11

**Authors:** Rodrigo E Mendes, Joshua Maher, Zachary Kockler, John H Kimbrough, Mariana Castanheira

**Affiliations:** Element Iowa City (JMI Laboratories), North Liberty, IA; Element Materials Technology/Jones Microbiology Institute, North Liberty, Iowa; Element Iowa City (JMI Laboratories), North Liberty, IA; Element Iowa City (JMI Laboratories), North Liberty, IA; Element, North Liberty, IA

## Abstract

**Background:**

Multidrug-resistant (MDR) *Acinetobacter baumannii-calcoaceticus* complex (ACB) have gained attention as an important clinical challenge in the last decades, due to its ability to develop resistance to front-line agents. Cefiderocol (FDC), a siderophore cephalosporin, uses the iron transport systems of Gram-negative bacteria to enhance cell entry. The activity of FDC and comparators was evaluated against ACB from US hospitals, including resistant subsets, collected as part of the SENTRY Antimicrobial Surveillance Program.

**Figure:**
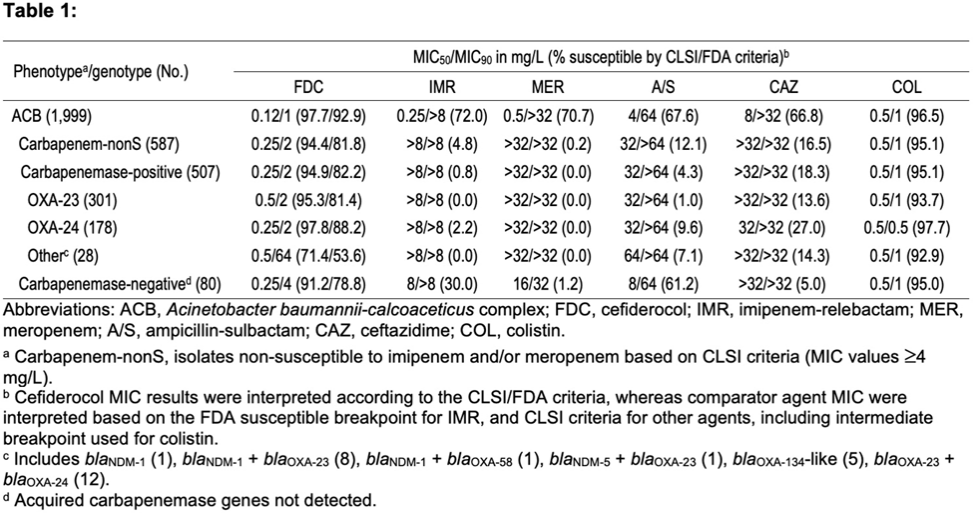

**Methods:**

1,999 ACB isolates were collected from 76 US sites (2020–2024). Susceptibility (S) testing was performed by broth microdilution with cation-adjusted Mueller-Hinton broth (CAMHB) for comparators and iron-depleted CAMHB for FDC. CLSI/FDA criteria were applied. Isolates with imipenem or meropenem MIC ≥4 mg/L (nonS by CLSI) were screened for β-lactamase genes.

**Results:**

29.4% (587/1,999) of isolates included were carbapenem-nonS, where carbapenemase genes were detected in 86.4% (507/587) of isolates (Table 1). *bla*_OXA-23_ (59.4%; 301/507) prevailed, followed by *bla*_OXA-24_ (35.1%; 178/507). A small number of isolates (5.5%; 28/507) carried mostly *bla*_NDM_ or double carbapenemases. FDC (92.9–97.7%S) had MIC_50_ of 0.12 mg/L and MIC_90_ of 1 mg/L against all ACB. β-lactam comparators had limited activity against these isolates (66.8–72.0%S). FDC (94.4–97.8%S by CLSI) had an MIC_90_ of 2 mg/L against carbapenem-nonS ACB carrying OXA-23- and OXA-24-like. A FDC MIC_90_ of 64 mg/L was obtained against the small subset carrying *bla*_NDM_ or double carbapenemases, with only ACB carying *bla*_NDM_ showing FDC MIC ≥2 mg/L. 13.6% (80/587) of carbapenem-nonS ACB did not carry acquired carbapenemases, and FDC (91.2%S; CLSI) was active against this subset.

**Conclusion:**

This study demonstrates the resistant nature of ACB causing infections in US hospitals, and FDC as an active agent against these isolates, regardless of carbapenem resistance phenotype or genotype. Other β-lactam agents, including newer and older β-lactam- β-lactamase-inhibitor combinations were inactive against this collection. These *in vitro* data suggest FDC as an important option for the treatment of infections caused by carbapenem-nonS ACB.

**Disclosures:**

Rodrigo E. Mendes, PhD, GSK: Grant/Research Support|Shionogi & Co., Ltd.: Grant/Research Support|United States Food and Drug Administration: FDA Contract Number: 75F40123C00140 Mariana Castanheira, PhD, Melinta Therapeutics: Advisor/Consultant|Melinta Therapeutics: Grant/Research Support

